# MiR-145 inhibits the differentiation and proliferation of bone marrow stromal mesenchymal stem cells by GABARAPL1 in steroid-induced femoral head necrosis

**DOI:** 10.1186/s12891-022-05928-z

**Published:** 2022-11-26

**Authors:** Pengfei Xu, Jun Chang, Guangwen Ma, Faxue Liao, Tangbing Xu, Yunfeng Wu, Zongsheng Yin

**Affiliations:** 1 Department of Orthopaedics, The First Affiliated Hospital of Anhui Medical University, 230000 Hefei, Anhui China; 2 Department of Orthopaedics, Anhui Public Health Clinical Center, 230000 Hefei, Anhui China

**Keywords:** Steroid-induced osteonecrosis of femoral head, microRNA-145, Bone marrow stromal mesenchymal stem cells, Proliferation, Differentiation, GABARAPL1

## Abstract

Steroid-induced osteonecrosis of femoral head (SANFH) involves impaired differentiation of bone marrow mesenchymal stem cells (BMSC), the mechanism of which is regulated by multiple microRNAs. Studies have shown that miR-145 is a key regulatory molecule of BMSC cells, but its mechanism in steroid-induced femur head necrosis remains unclear. The present study mainly explored the specific mechanism of miR-145 involved in SANFH. In this study dexamethasone, a typical glucocorticoid, was used to induce osteogenic differentiation of BMSC cells. Western blot, qPCR, CCK8 and flow cytometry were used to investigate the effects of miR-145 on the proliferation and differentiation of BMSC. The relationship between miR-145 and GABA Type A Receptor Associated Protein Like 1(GABARAPL1) was identified using dual luciferase reports and the effects of the two molecules on BMSC were investigated in vitro. The results showed that miR-145 was up-regulated in SANFH patients, while GABARAPL1 was down-regulated. Inhibition of miR-145 can improve apoptosis and promote proliferation and activation of BMSC. GABARAPL1 is a downstream target gene of miR-145 and is negatively regulated by miR-145. In conclusion, miR-145 regulates the proliferation and differentiation of glucocorticoid-induced BMSC cells through GABARAPL1 and pharmacologically inhibit targeting miR-145 may provide new aspect for the treatment of SANFH.

**Supplementary Information:**

The online version contains supplementary material available at 10.1186/s12891-022-05928-z.

## Introduction

Steroid-induced osteonecrosis of femoral head (SANFH) is a common clinical disease which is a kind of metabolic disease of bone mainly due to the high dose of glucocorticoid [1]. Improper use of glucocorticoids leads to the disturbance of bone circulation and the imbalance of bone metabolism, and then destroys the local blood supply of the femoral head and causes bone tissue ischemic necrosis, eventually leading to the collapse and fracture of the femoral head [2]. With the widespread use of glucocorticoids in clinical practice, about 40% of users end up with osteonecrosis, among which patients with SANFH is the most common [3]. After the onset of femoral head necrosis, the condition is difficult to reverse. Due to its insidious onset, rapid development and strong disablement, the disease has become a major public problem troubling the society. However, as for the pathogenesis mechanism of SANFH, there is still no theory that can fully explain.

In recent years, mesenchymal stem cells (MSCs) has become an intense focus of cell-based therapies for various diseases because of the regenerative capacity and immunomodulatory properties. The International Society of Cell Therapy (ISCT) identifies MSCs by three criteria. Bone marrow mesenchymal stem cells (BMSCs) are a group of stem cells with multidirectional differentiation ability that can differentiate into a variety of cells including osteoblasts in a specific microenvironment [4–7]. Previous studies have shown that changes in osteogenic differentiation ability of bone marrow mesenchymal stem cells are closely related to SANFH [8]. It has been confirmed that the osteogenic differentiation ability of BMSCs decreased in patients with SANFH, but the adipose differentiation ability increased [9]. In addition, proliferation of BMSCs was significantly reduced in patients with SANFH [10]. These results suggest that BMSCs are key regulatory cells of SANFH. Therefore, finding molecules that regulate BMSCs proliferation and osteoblast differentiation is critical to identify new targets for SANFH prevention.

MicroRNA can regulate cell metabolism and development, promote cell proliferation and differentiation, and regulate cell apoptosis by regulating the expression of certain signal molecules in cell signals, such as transcription factors, cytokines, growth factors, and pro-apoptotic and anti-apoptotic genes [11–13]. With the deepening of microRNA research, scholars have extended microRNA to the related research of some diseases, such as the occurrence and development of tumor and the pathogenesis of autoimmune diseases, etc. [14, 15]. Meanwhile, the research in the field of orthopedics is also deepening with time, for example, some microRNA has been confirmed to participate in and regulate bone tissue metabolism and chondrocyte formation [16]. Although the specific regulatory mechanism of miRNAs has not been fully elucidated, the regulatory importance of miRNAs in bone, cartilage and chondrocytes is beyond doubt [17]. Studies have shown that microRNA plays an important role in the post-transcriptional regulation of BMSCs [18], and the differentiation of BMSCs can also cause a variety of miRNA up- or down-regulation. For example, the expression of miR-199a was more than 10 times higher in chondroblasts than in undifferentiated BMSCs. miR-199 also regulates BMSC differentiation by targeting the expression of human hypoxia-inducible factor (HIF-1α) [19]. A recent study showed that the down regulation of miR-96 expression can effectively promote the directed differentiation of BMSC cells into osteoblasts, possibly by regulating the expression of Sox9 protein at the post-transcriptional level [20]. Inducing osteogenic differentiation of BMSC is also a key target for treatment of SANFH. However, whether miR-145 is involved in SANFH by regulating the function of BMSC and how it works have not been reported at home and abroad. GABARAPL1 was considered as downstream molecule of some other microRNAs like miR-133a-3p [21] and mi R-15a-5p [22]. It was reported that GABARAP (ATG8) subfamily proteins served as scaffolding proteins by recruiting ULK1 and beclin-1 (complex) to the site of autophagosome nucleation and directly participant autophagy more efficiently than LC3. So we checked GABARAPL1 as a downstream target gene of miR-145. The purpose of this study was to explore the role and related mechanism of miR-145 in SANFH, and to provide a new theoretical basis for the targeted treatment of steroid-induced femur head necrosis.

## Methods

### Patients

Serum samples from a different set of 21 SANFH and 21 non- SANFH patients were detected miR-145 expression in the samples by qPCR, which were obtained from The First Affiliated Hospital of Anhui Medical University. The normal controls were qualified blood donors and all of them have no evidence of SANFH. The Ethics Commission of The First Affiliated Hospital of Anhui Medical University approved this study, with a waiver of informed consent.

### BMSC culture and transfection

Human BMSCs were purchased from the Procell Life Science & Technology (Wuhan, China). High-glucose Dulbecco’s modified Eagle (DMEM) medium with 10% fetal bovine serum (FBS) and 1% penicillin-streptomycin (PS) were used to culture hBMSCs (DMEM: Hyclone, Logan, UT, USA, FBS: Gibco, Carlsbad, CA, USA, PS: Gibco, Carlsbad, CA, USA ). Ccll media was replaced every 3 days. Passage was carried out when the flask was 75% full. miR-145 mimic, mimic control, inhibitor, inhibitor control and GABARAPL1 sh-NC and sh-GABARAPL1 were diluted and packaged separately. BMSCs were selected for slab laying the night before the transfection experiment. Adjust the cell count to 7 × 10^6^. During the experiment, the expression vector was diluted with opti-MEM (ThermoFisher, USA)and mixed with the corresponding cells. The cells were gently mixed and incubated at room temperature for 20 min. Then cells were washed at least twice with serum-free DMEM medium, and then added serum-free DMEM medium to the cells and cultured in incubator for 6 h. After 5 h, the serum-free medium was discarded and DMEM medium containing 10%FBS was added to continue culture. The transfection was checked 18 h later, and the transfected cells were used for subsequent experiments [23, 24].

### Western blot

The expression of apoptosis-related protein、Osteogenic associated proteins and GABARAPL1 were examined by western blot as previously described. Proteins were extracted from BMSC cells. Bicinchoninic acid (BCA, Beyotime) kit were used to determine the concentration of the protein with a standard by bovine serum albumin (BSA). Adjusting all protein samples to equal concentrations and diluted with 5× loading buffer and stored in -80 °C refrigerator. Before western bolt experiment, denaturing all samples at 100 °C for 10 min. The same amount of total protein samples were separated by electrophoresis in 8% sodium dodecyl sulfate polyacrylamide gel (SDS-PAGE), and then the gel was transferred to a polyvinylidene fluoride membrane (Millipore, Billerica, MA, USA). Put immunoblots in 5% skim milk diluting by Trisbuffered saline/Tween-20 buffer (TBST) for 1 h for block. Then the primary antibodies (Bax, Bcl-2, cleaved-caspase3, cleaved-caspase9, β-actin, ALP, RUNX2, OPN, BMP2, GABARAPL1) were incubated at 4 °C overnight (Proteintech, Chicago, USA,1:1000) [25–28]. Secondary antibody were incubated at room temperature for 1 h (1:1000 dilution; Beijing Biosynthesis Biotechnology, Beijing, China). The antibodies are shown at Table 1. Quantity One one-dimensional analysis software (Bio-Rad) were used to analyses the results.

**Table 1:** Antibodies used in study

Name	Catalog number	Sourse	Company
primary antibody			
Bax	50599-2-Ig		Proteintech, Chicago, USA
Bcl-2	26593-1-AP		
cleaved-caspase3	19677-1-AP		
β-actin	20536-1-AP		
ALP	18507-1-AP		
RUNX2	20700-1-AP		
OPN	22952-1-AP		
BMP2	155,441-AP		
GABARAPL1	18721-1-AP		
Secondary antibody	bs-0312R		Beijing Biosynthesis Biotechnology

### Quantitative polymerase chain reaction

miR-145 purification kit was purchased from EZ Bioscience, Beijing, China and the cDNA of miR-145 were extracted by microRNA Reverse Transcription Kit (EZ Bioscience, Carlsbad, USA). The amplification of miR-145 was carried out by 2× qPCR Mix for microRNA (EZ Bioscience, Carlsbad, USA). GAPDH was used to normalized the expression of miR-145. The primers listed as below:

U6:

forward primer: CTCGCTTCGGCAGCACA.

reverse primer: AACGCTTCACGAATTTGCGT.

miR145: forward primer: GTCCAGTTTTCCCAGGAA.

reverse primer:CAGGTCAAAAGGGTCCTT.

GABARAPL1: forward primer: ATGAAGTTCCAGTACAAGGAGGA.

Reverse primer: GCTTTTGGAGCCTTCTCTACAAT.

### Cell counting kits for proliferation assay

Cell counting kits (CCK-8, Dojindo, Kumamoto, Japan) were used to estimate the activity and ability of proliferation of BMSCs in different stimulation. The cells were seeded in 96-well plates at a density of 5000 cells per well. Culturing cells with the following different treatments: control, 10 µM dexamethasone (DEX; Solarbio, Beijing, China), DEX + NC inhibitor, DEX + miR-145 inhibitor, DEX + NC inhibitor + sh-NC, DEX + miR-145 inhibitor + shGABARAPL1(MedCemExpress, USA). OD values OD values were read and cell activity was analyzed.

### Alizarin red staining and alkaline phosphatase (ALP) activity

After cell density reached 60–70%, the cells were changed to osteogenic differentiation medium (Cyagen, Guangzhou, China) to promote differentiation. Then 10 µM DEX, CD34+-Exos (50 µg/mL), or miR-26a-CD34+-Exos (50 µg/mL) were added to the BMSC cultures. Alizarin red staining (Cyagen) was performed on day 21 after incubation. Cells were washed three times in PBS and fixed in 4% paraformaldehyde for 15 min. Alizarin red staining was used to determine osteogenic activity. ALP activity was evaluated using a BCIP/NBT Alkaline Phosphatase Color Development Kit (Solarbio, Beijing, China) on day 7 of incubation. The images were acquired using a light microscope (Olympus IX 70). After the Alizarin red staining was observed by the microscope, the stained calcium nodules were eluted by 10% cetyplyridinium chloride for 1 h, and the absorbance of the solution was read on the microplate reader at 550 nm [29].

### Flow cytometry

Mature BMSC cells were collected for cell count. 1 ~ 5 × 10^6^ cells were taken into the centrifuge tube and centrifuged for 5 min (1000r/min). After discarding the culture medium, the cells were cleaned with PBS once. Add 70% iced ethanol and set at 4 ° C for 1 h. The fixation solution was then discarded and the cells were resuspended with PBS solution. Add PI and Annexin V (Signalway Antibody, CA004, USA) dye to the cells. Apoptosis could be detected after 30 min in 4 °C with light avoidance staining.

### Double luciferase assay

All target genes of miR-145 were predicted using miRbase online software, and the predicted target genes were analyzed using DAVID online software. In this study, a luciferase reporter vector and its mutant vector of GABARAPL1 gene 3’-UTR terminal were constructed. GABARAPL1 3′UTR sequence containing wild type or mutant miR-145 putative binding region was amplified by RiboBio (Shanghai, China) and inserted into pGL3-GP73-3′UTR plasmid (Invitrogen, USA). Plasmid were then transfected into BMSC cells and divided into GABARAPL1 WT + NC mimic group, GABARAPL1 WT + miR-145 mimic group, GABARAPL1 Mut + NC mimic and GABARAPL1 Mut + miR-145 mimic group. Lipofectamine 2000 (Invitrogen, USA) was used to co-transfected. 48 h after transfection, the cell culture medium was sucked out. Add 75µL PBS and 75µL luciferase substrate to each well. Shock in the dark for 10 min. The fluorescence value of luciferase was measured with Dual-Luciferase Reporter Assay System (Promega, USA).

### Statistical analysis

Measurement data are represented by means. T test was used to compare the mean values of two different groups when the values followed a normal distribution. If the values did not follow a normal distribution the *Mann*-*Whitney* U *test* was considered. One-way or two-way analysis of variance (ANOVA) were used to compare the mean values of measurement data of multiple groups. All tests should be repeated at least 3 times. All data were recorded in excel and counted in SPSS24.0. *P* values < 0.05 were considered statistically significant.

## Results

### Serum miR-145 expression is up regulated in patients with SANFH

**Fig. 1:**
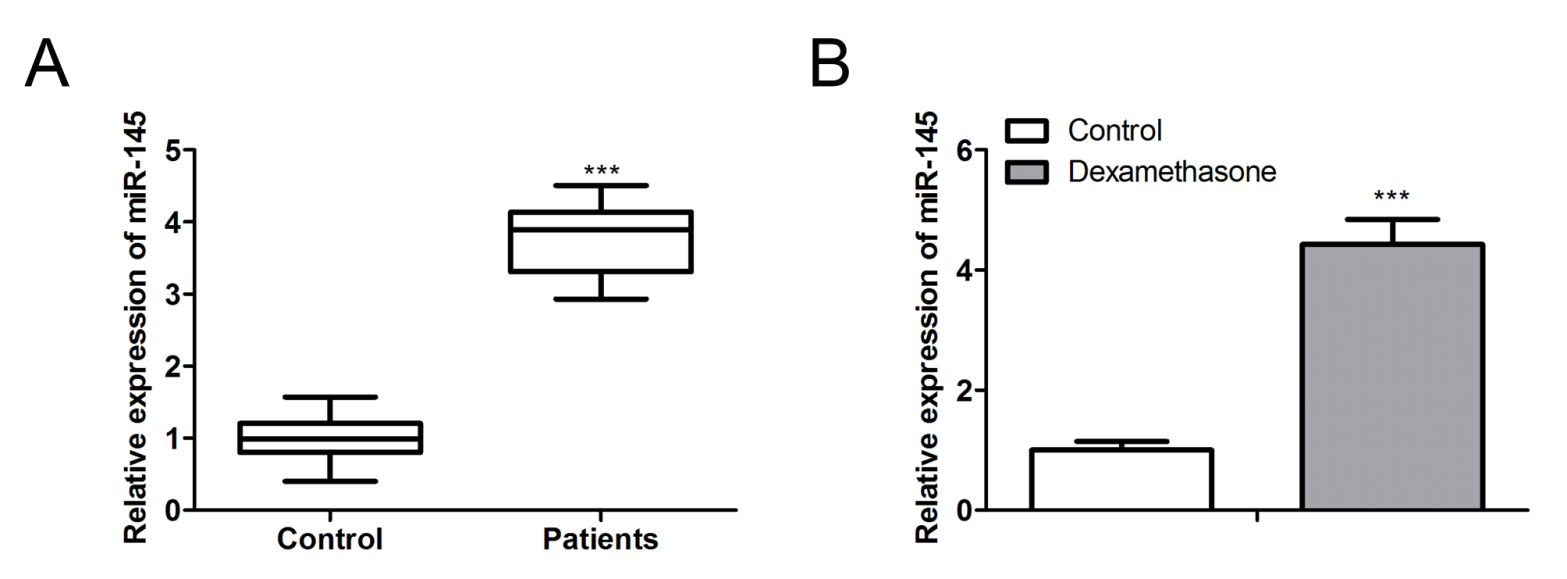
Serum miR-145 up regulated in patients with SANFH. (A). The expression of miR-145 in serum of 21 SANFH and 21 non- SANFH patients were detected by qPCR. (B). qPCR detected the expression of miR-145 in DEX induced BMSCs (***P < 0.001)

We obtained serum samples from a different set of 21 SANFH and 21 non- SANFH patients and detected miR-145 expression in the samples by qPCR. The result is shown in Fig. 1A. The expression of miR-145 in patients with femoral head necrosis was 4 times higher than that in control group. Meanwhile, primary BMSC cells were cultured in vitro and the expression of miR-145 was detected after 24 h stimulation of dexamethasone (DEX). The results showed that dexamethasone stimulation significantly up-regulated the expression of miR-145 in cells (Fig. 1B).

### miR-145 regulates apoptosis of BMSCs caused by glucocorticoid

**Fig. 2:**
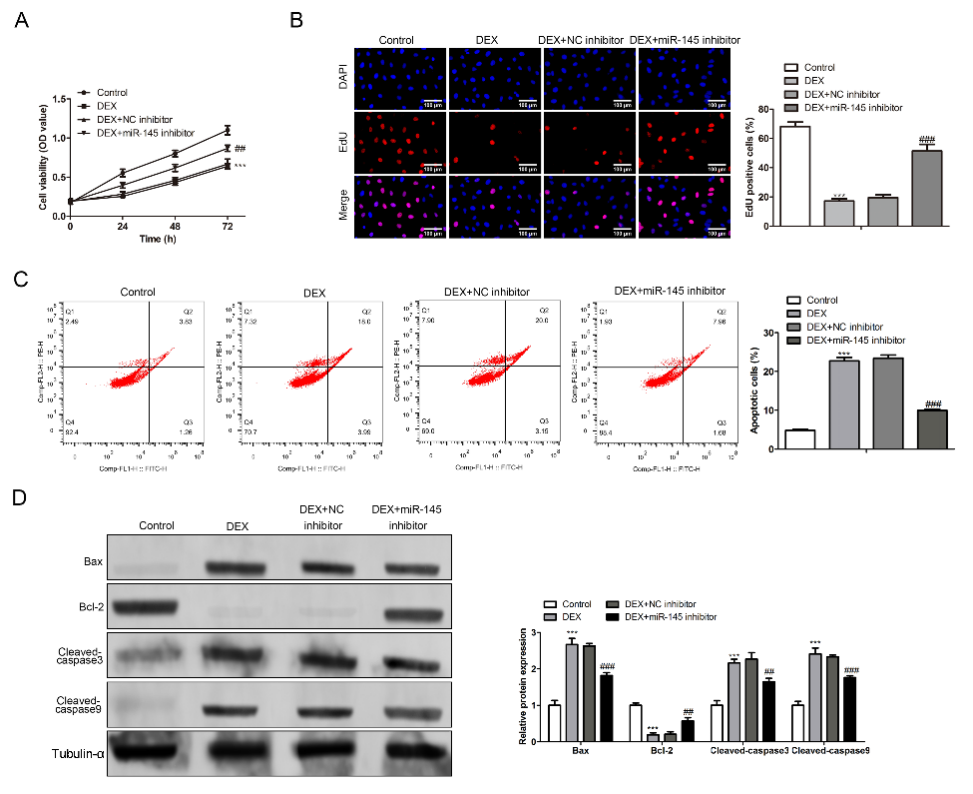
miR-145 regulates apoptosis of BMSCs caused by glucocorticoid. (A) CCK8 detected the cell activity of BMSC cells in different groups. (B) The proliferation of BMSC was detected by EdU immunofluorescence staining. (C) Apoptosis of BMSC cells was detected by flow cytometry. (D) The expression of apoptosis-related proteins (Bcl, Bax, cleaved-caspase3 and cleaved-caspase 9) was detected by western blot (Scale bar: 100 μm, DEX vs. control, ***P < 0.001, DEX + miR-145 inhibitor vs. control, ##P < 0.01, ###P < 0.001)

In this part we determined the effect of miR-145 on apoptosis of BMSC after glucocorticoid stimulation in vitro. BMSC cells at logarithmic stage were divided into control group, DEX stimulation group, DEX + NC inhibitor group and DEX + miR-145 inhibitor group according to different intervention conditions. CCK8 results showed that the activity of BMSC cells significantly decreased after DEX stimulation, and there was no statistical difference between the activity of BMSC cells and that of DEX + NC inhibitor group. While the activity of BMSC cells significantly increased after inhibiting the activity of miR-145 (Fig. 2A). The proliferation ability of each group was detected by EdU staining. The results showed that the proliferation level of BMSC decreased 4 times after DEX stimulation, while the proliferation level was significantly increased after the addition of miR-145 (Fig. 2B). At the same time, flow cytometry was used to further evaluate the apoptosis of miR-145. The results were similar to CCK8. DEX stimulation led to a significant increase in the proportion of apoptotic cells, while the trend was significantly reversed when adding miR-145 inhibitors (Fig. 2C). Finally, we used western blot to detect the expression level of apoptosis-related proteins. The results showed that protein content of Bax, cleaved-caspase3 and cleaved caspase 9 was significantly increased after DEX stimulation, and their expression decreased after miR-145 inhibitor stimulation. The difference was statistically significant. The expression trend of Bcl-2 protein was the opposite as expected (Fig. 2D).

### miR-145 regulates differentiation of BMSCs caused by glucocorticoid

**Fig. 3:**
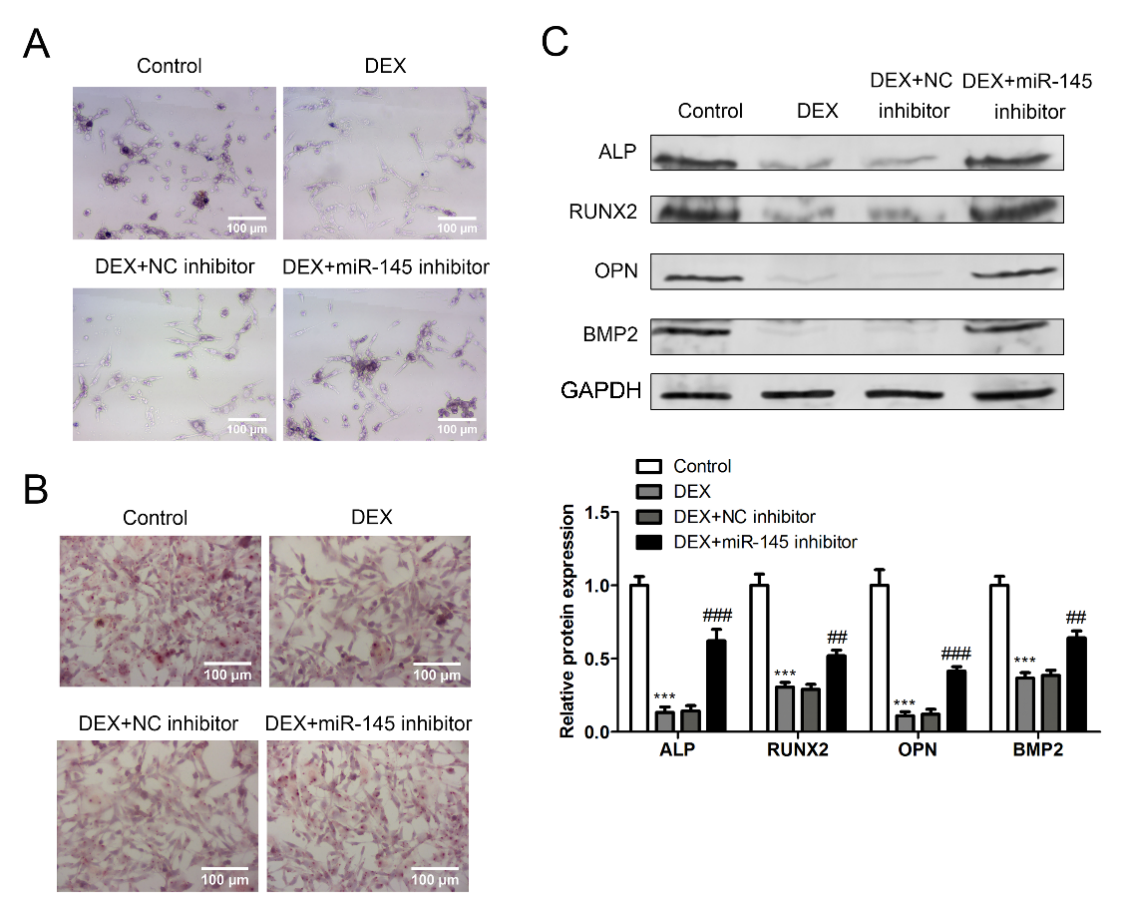
miR-145 regulates differentiation of BMSCs caused by glucocorticoid. (A) ALP staining was used to determine the expression of ALP in different groups. Scale bar: 100 μm. (B) Formation of calcium nodules was analyzed by Alizarin red staining in the different groups. Scale bar: 100 μm. (C) The expression of differentiation-related proteins (ALP, RUNX2, OPN, BMP2) in BMSC was detected by western blot (DEX vs. control, ***P < 0.001, DEX + miR-145 inhibitor vs. control, ^###^*P* < 0.001)

Then, the effect of miR-145 on BMSC differentiation after glucocorticoid stimulation in vitro. ALP staining can be used to observe the morphology of osteoblasts and osteoclasts to determine the differentiation state of bone tissue. The results showed that inhibition of miR-145 expression could save the abnormal differentiation of BMSC induced by DEX stimulation, that is, the expression of ALP in miR145 inhibitor group was higher and more significant (Fig. 3A). Moreover, the formation of calcareous nodules was also observed by alizarin red staining. As is shown in Fig. 3B, the number of calcareous nodules in DEX stimulated BMSC cells was significantly reduced, which was reversed by inhibiting miR-145 expression. Finally, we also examined the expression of osteogenic related protein ALP, RUNX2, OPN and BMP2. The results showed that the expression of all osteogenic proteins in BMSC was significantly decreased by DEX stimulation, while the expression of these proteins was significantly increased by inhibiting the expression of miR-145 (Fig. 3C). These results suggest that miR-145 is closely related to the differentiation process of BMSC.

### miR-145 targets downstream GABAPARL1 expression

**Fig. 4:**
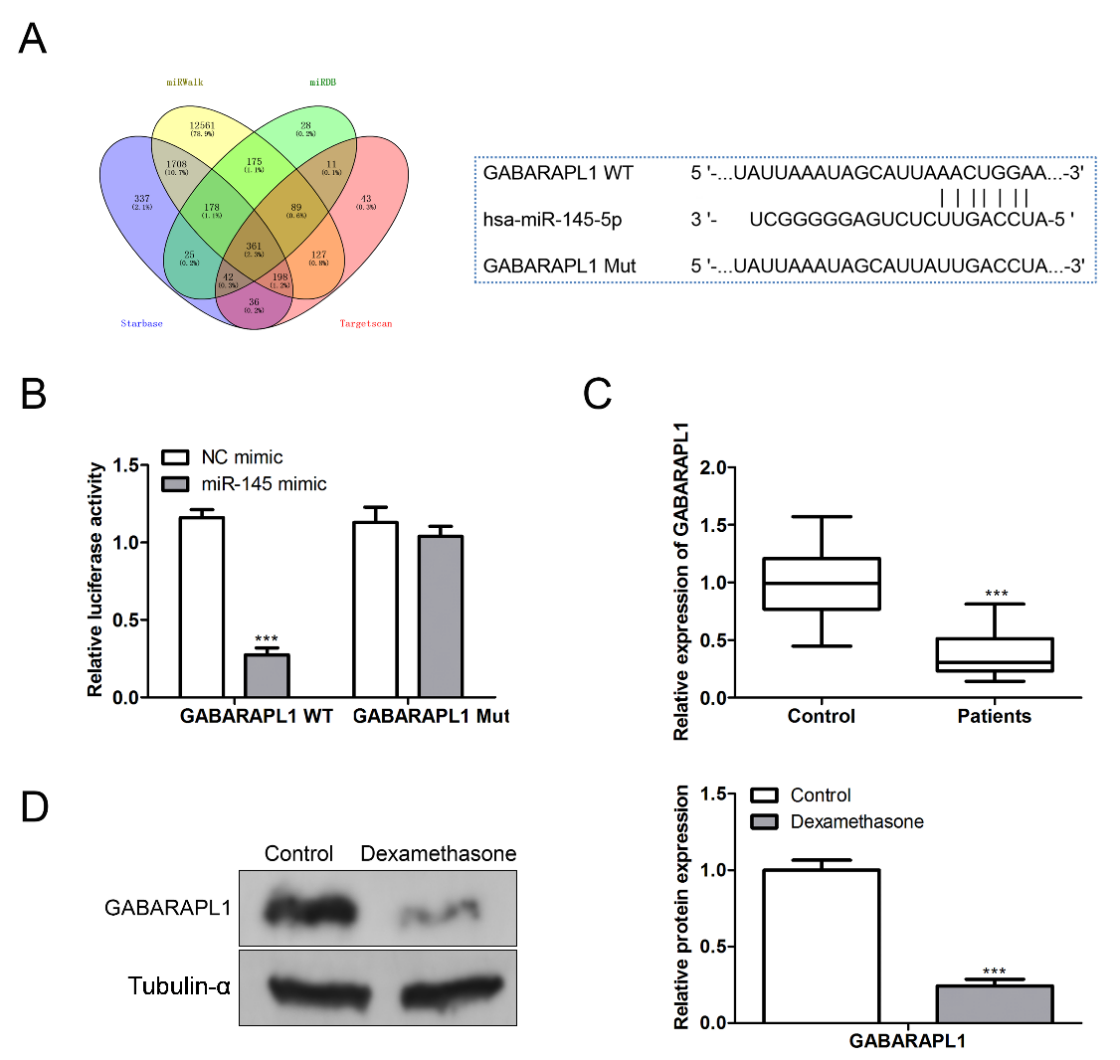
miR-145 targets downstream GABARAPL1 expression. (A)Double luciferase report of miR-145 targeting GABARAPL1. (B) Detection of luciferase activity in cells transfected with miR-145 mimic. (C) The expression of GABARAPL1 in serum of SANFH patients detected by qPCR. (D) The expression of GABARAPL1 in DEX induced cells was detected by western blot (***P < 0.001)

Next, we further explored the downstream targets of miR-145. Among the predicted downstream regulatory genes of miR-145, GABARAPL1 is associated with SANFH. First, double luciferase assay was used to verify the targeting effect of miR-145 and GABARAPL1 (Fig. 4A, B). The analysis report showed that GABARAPL1 expression was significantly decreased under the action of miR-145. However, when GABARAPL1 gene is mutated, miR-145 no longer regulates its expression, suggesting the targeted regulation of miR-145 on GABARAPL1. Subsequently, we investigated whether GABARAPL1 is involved in the pathogenesis of SANFH femoral head necrosis. First, we detected the expression of GABARAPL1 in serum of clinical patients. The results showed that GABARAPL1 expression was lower in serum of patients with SANFH than in the normal population, and the difference was statistically significant (Fig. 4C). Then, the expression of GABARAPL1 was further detected in vitro experiments. Western blot results showed that the expression of GABARAPL1 protein was significantly reduced in DEX stimulated BMSC cells (Fig. 4D).

### miR-145 regulates glucocorticoid-induced apoptosis and differentiation of BMSC by down-regulating GABARAPL1 expression

**Fig. 5:**
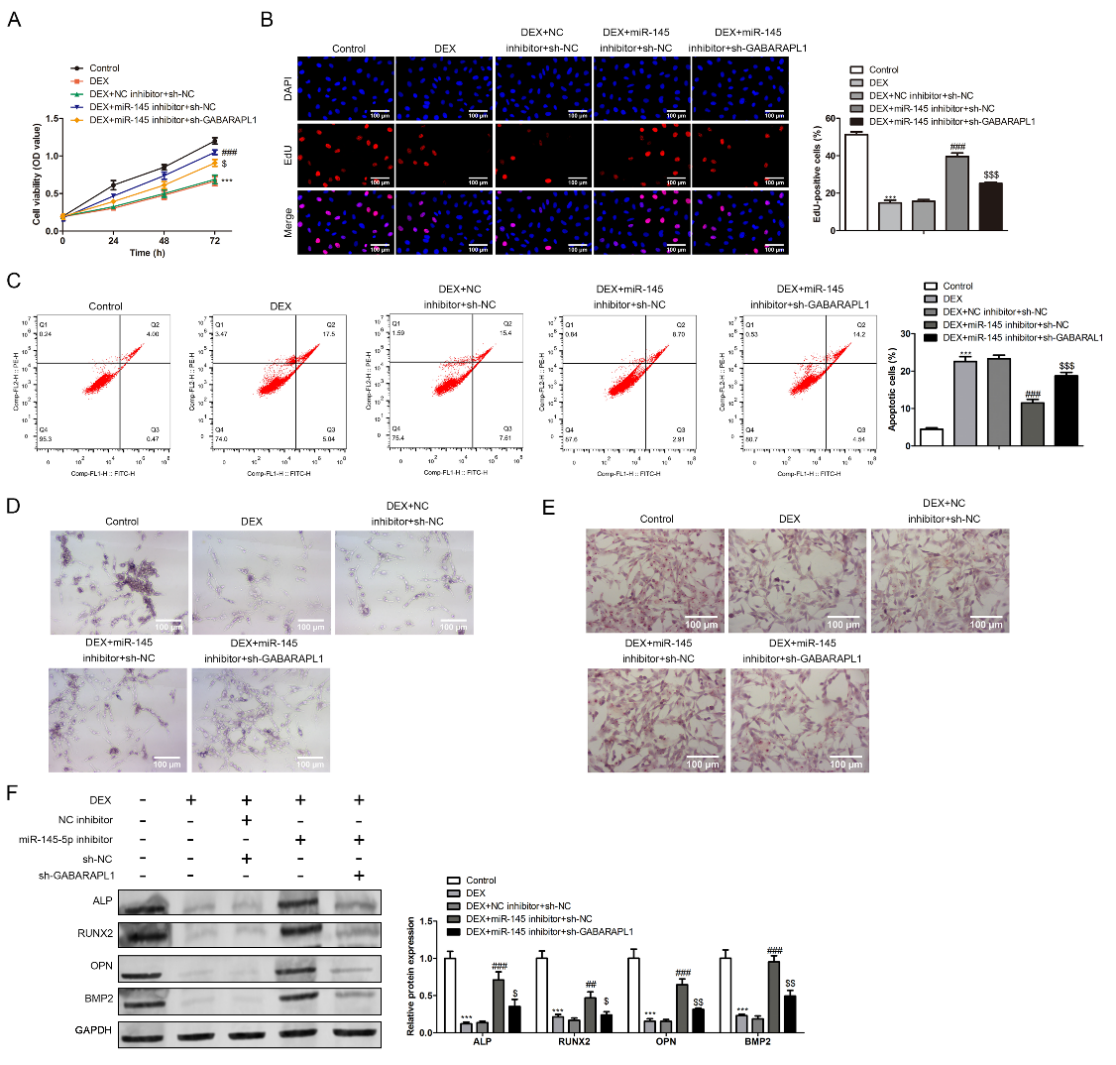
miR-145 regulates glucocorticoid-induced apoptosis and differentiation of BMSC by down-regulating GABARAPL1 expression. (A)CCK8 assayed the activity of different groups of cells. (B) The proliferation level of BMSC cells was detected by EdU immunofluorescence double staining. (C) Flow cytometry was used to detect the apoptosis of cells in different groups. (D, E) The differentiation of BMSC cells was detected by ALP and alizarin red staining. (F) The expression of differentiation-related proteins (ALP, RUNX2, OPN, BMP2) in different groups were detected by western blot (Scale bar: 100 μm, DEX vs. control, ***P < 0.001, DEX + miR-145 inhibitor + sh-NC vs. control, ^##^*P* < 0.01, ^###^*P* < 0.001, DEX + miR-145 inhibitor + sh-GABARAPL1 vs. control, ^$^*P* < 0.05, ^$$^*P* < 0.01, ^$$$^*P* < 0.001)

Finally, we conducted a set of rescue experiments to explore the specific mechanism of miR-145 involved in SANFH injury by regulating GABARAPL1 expression. Mature BMSC cells were divided into control, DEX, DEX + NC inhibitor + sh-NC, DEX + miR-145 inhibitor + sh-NC and DEX + miR-145 inhibitor + sh-GABARAPL1 group. CCK8 assay was first used to detect changes in cell activity under different stimulation conditions (Fig. 5A). As indicated above, DEX stimulation down-regulated cell activity, and inhibition of miR-145 expression up-regulated cell activity. However, if the expression of GABARAPL1 was interfered while miR-145 was suppressed, the cell activity would be decreased. EdU and flow cytometry showed that inhibition of miR-145 expression improved apoptosis induced by DEX (Fig. 5B and C). While, when GABARAPL1 expression was interfered the proportion of apoptotic cells increased. Next, the effects of miR-145 and GABARAPL1 on the differentiation level of BMSC cells were further investigated by ALP and alizarin red detection. The results showed that ALP expression was more significantly and the content of calcium nodules was higher after miR-145 inhibition. However, the expression of osteogenic markers in BMSC decreased after GABARAPL1 expression was inhibited (Fig. 5D and E). At last, we detected the expression of osteogenic related proteins in each group of cells to further determine the effect of miR-145 and GABARAPL1 on the differentiation ability of BMSC. Results showed that the expression of osteogenic associated protein ALP was elevated in the presence of miR-145 inhibition, but interference with GABARAPL1 reversed this trend (Fig. 5F).

## Discussion

In this study, we found that serum miR-145 expression was significantly up-regulated in patients with clinical steroid-induced femur head necrosis. The regulation of miR-145 on apoptosis and differentiation was confirmed by a series of in vitro experiments targeting BMSCS. GABARAPL1, the downstream target gene of miR-145, is also involved. This is the first study to investigate whether miR-145 plays a role in the development of SANFH. Our results suggest that miR-145 and its downstream pathway molecules may be effective therapeutic targets for femur head necrosis induced by glucocorticoid.

Osteoblast differentiation block is one of the main causes of SANFH [30]. It includes two aspects: the slowing down rate of osteoblast differentiation and reduced number of osteoblast differentiation [31]. In order to improve the therapeutic effect of SANFH, enough attention has been paid to the regulation of this process. BMSCs are important stromal cells that regulate bone tissue development and metabolism. Previous studies on BMSC and SANFH showed that the ability of BMSC to differentiate into osteoblasts was weakened and the ability to differentiate into adipose tissue was enhanced when SANFH occurred [32]. Promoting osteogenic differentiation of BMSC can alleviate bone loss in SANFH rats [33]. Based on the above experimental results, it can be concluded that the differentiation of BMSC is an important cytological basis of SANFH regulation, and the exploration of the molecular mechanism of BMSC cell differentiation is expected to provide a new direction for the treatment of SANFH. As endogenous non-coding small molecule RNA, microRNA has a variety of functions and mechanisms of action. For example, it can cause target mRNA degradation by specifically binding target genes, and it can also inhibit protein translation, regulate gene transcription, disease occurrence and development as well as body development process [34]. Most importantly, miRNA is also actively involved in the proliferation, differentiation and apoptosis of somatic cells. Research data indicate that miRNA can affect the proliferation and differentiation of BMSCs through different signaling pathways [35], but the role of miRNA in steroid-induced femoral head necrosis remains to be further studied. miR-145 is a microRNA involved in the immune process, but there are few studies on its downstream regulatory relationship. Furthermore, its role in SANFH has not been reported. In the present study, we found that the expression of miR-145 in serum of SANFH patients was significantly increased clinically, suggesting that miR-145 may be involved in the progression of SANFH. Through in vitro glucocorticoid stimulation, we found that miR-145 expression also showed an up-regulated trend. After determining the variation trend of miR-145 in SANFH, we further explored the regulatory effect of miR-145 on BMSC cells in vitro. The results showed that the activity and proliferation of BMSC cells were decreased and the apoptosis level was increased after DEX stimulation. The expression level of apoptosis-related proteins was also significantly increased. However, these changes can be reversed with miR-145 inhibitors. In other words, inhibition of miR-145 expression can promote the proliferation and activity of DEX stimulated BMSC cells and alleviate the continuous progression of apoptosis. Not only that, miR-145 also has a direct regulatory effect on the osteogenic differentiation of BMSCs. Inhibiting the expression of miR-145 can significantly alleviate the delay in differentiation induced by DEX and promote the normal differentiation of BMSC cells into osteoblasts. Therefore, we speculate that the change of miR-145 content is also one of the key molecular mechanisms leading to the pathogenesis of SANFH.

Several studies on microRNA in the past have focused on the regulation of the proliferation and differentiation of BMSC and confirmed that these regulatory effects of microRNA indeed play an important role in the development of SANFH [36, 37]. For example, miR-596 can inhibit osteoblast differentiation through Smad3 molecule [38], and miR-141 can inhibit the proliferation of BMSC through SOX11 [39], etc. Our results are similar with these studies, but our data show that miR-145 can simultaneously regulate the differentiation, apoptosis and proliferation of BMSC. Besides, it has been reported that miR-145 suppresses osteogenic differentiation of human jaw bone marrow mesenchymal stem cells partially via targeting semaphorin 3 A, which showed a different effect of miR-145 in some other disease model [40]. To further clarify the effect of miR-145 in SANFH, we designed the following experiment to demonstrate the role of it. Considering the lack of studies on downstream targets of miR-145, we further expanded our research on GABARAPL1, its downstream target molecule. Our experimental results showed that GABARAPL1 was also involved in the progress of SANFH under the regulation of miR-145. This is of great concern. While miR-145 was up-regulated in serum of SANFH patients, GABARAPL1 expression was significantly down-regulated. The above results are even more noteworthy after the targeting relationship between the two was confirmed by dual luciferase assay. The results of rescue experiment confirmed that the status of BMSC after DEX stimulation was not only affected by miR-145, but also closely related to the expression of GABARAPL1. In other words, inhibition of miR-145 expression can promote cell proliferation and reduce apoptosis. However, inhibition of GABARAPL1 expression reverses this process. These results indicate the targeting relationship of miR-145 and GABARAPL1 and suggest the potentially important role of this signaling pathway in SANFH.

As important regulatory molecules in physiological and pathological, microRNA have received increasing attention for their functions in diseases. We selected miR-145 as the object of this study to explore the role of miR-145 in SANFH for the first time. Through this study, we confirmed the up-regulated expression of miR-145 in clinical SANFH patients. This result also supports us to further explore the effects of miR-145 through in vitro experiments. At the same time, this study also identified the downstream signaling molecules of miR-145 and improved the mechanism of miR-145 in SANFH. However, this study did not carry out relevant verification from animal experiments, nor did more detailed in vivo experiments to prove the role of miR-145. Subsequent studies should further complement the mechanism of miR-145 through in vivo experiments. Last but not least, the results of in vitro experiments can also fully suggest the therapeutic potential of miR-145 in SANFH. The possibility of treating SANFH with this molecule can be further explored in the future.

## Electronic supplementary material

Below is the link to the electronic supplementary material.

Supplementary Material 1

## Data Availability

The raw data supporting the conclusions of this manuscript will be made available by Dr. Zongsheng Yin, without undue reservation, to any qualified researcher.
