# Supplementary material for: MiR-145 inhibits the differentiation and proliferation of bone marrow stromal mesenchymal stem cells by GABARAPL1 in steroid-induced femoral head necrosis
Source: BMC Musculoskelet Disord. 2022 Nov 26;23:1020. doi: 10.1186/s12891-022-05928-z (PMC9701430; doi:10.1186/s12891-022-05928-z)
Supplement: Supplementary file 1 — Supplementary Material 1 [file 12891_2022_5928_MOESM1_ESM.pptx]

## Slide 1
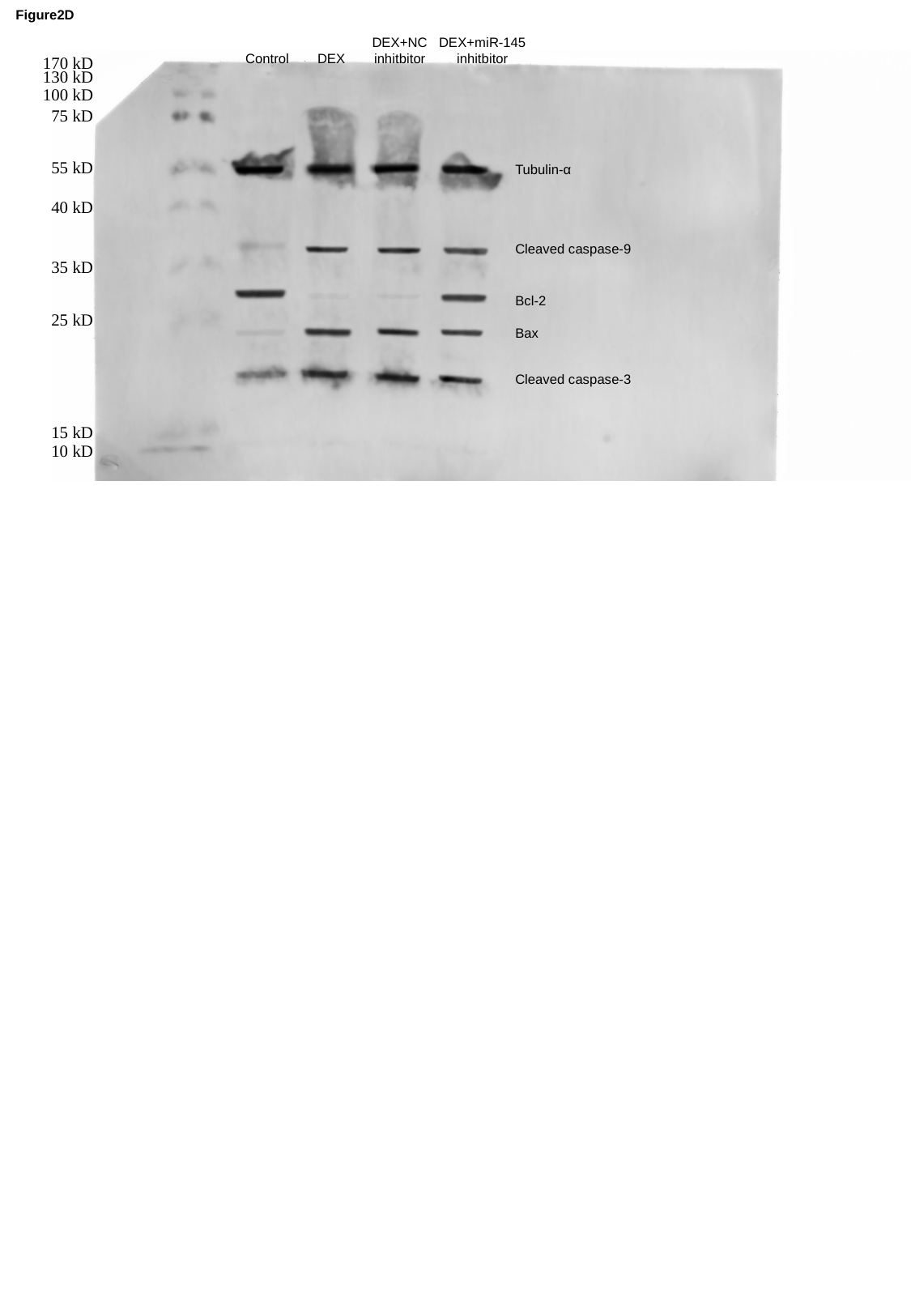

Figure2D
DEX+NC inhitbitor
DEX+miR-145 inhitbitor
Control
DEX
170 kD
130 kD
100 kD
75 kD
55 kD
Tubulin-α
40 kD
Cleaved caspase-9
35 kD
Bcl-2
25 kD
Bax
Cleaved caspase-3
15 kD
10 kD

## Slide 2
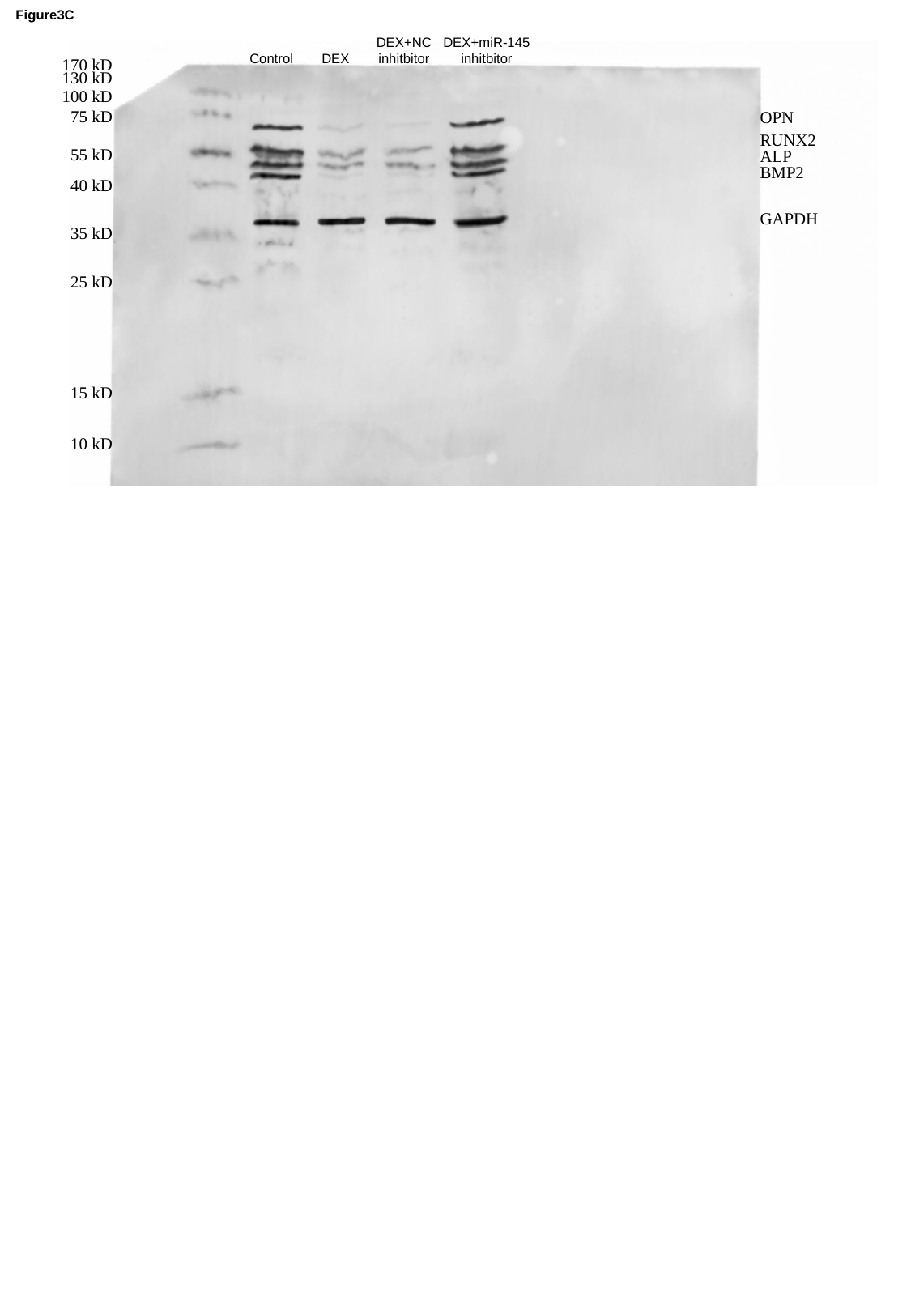

Figure3C
DEX+NC inhitbitor
DEX+miR-145 inhitbitor
Control
DEX
170 kD
130 kD
100 kD
75 kD
OPN
RUNX2
55 kD
ALP
BMP2
40 kD
GAPDH
35 kD
25 kD
15 kD
10 kD

## Slide 3
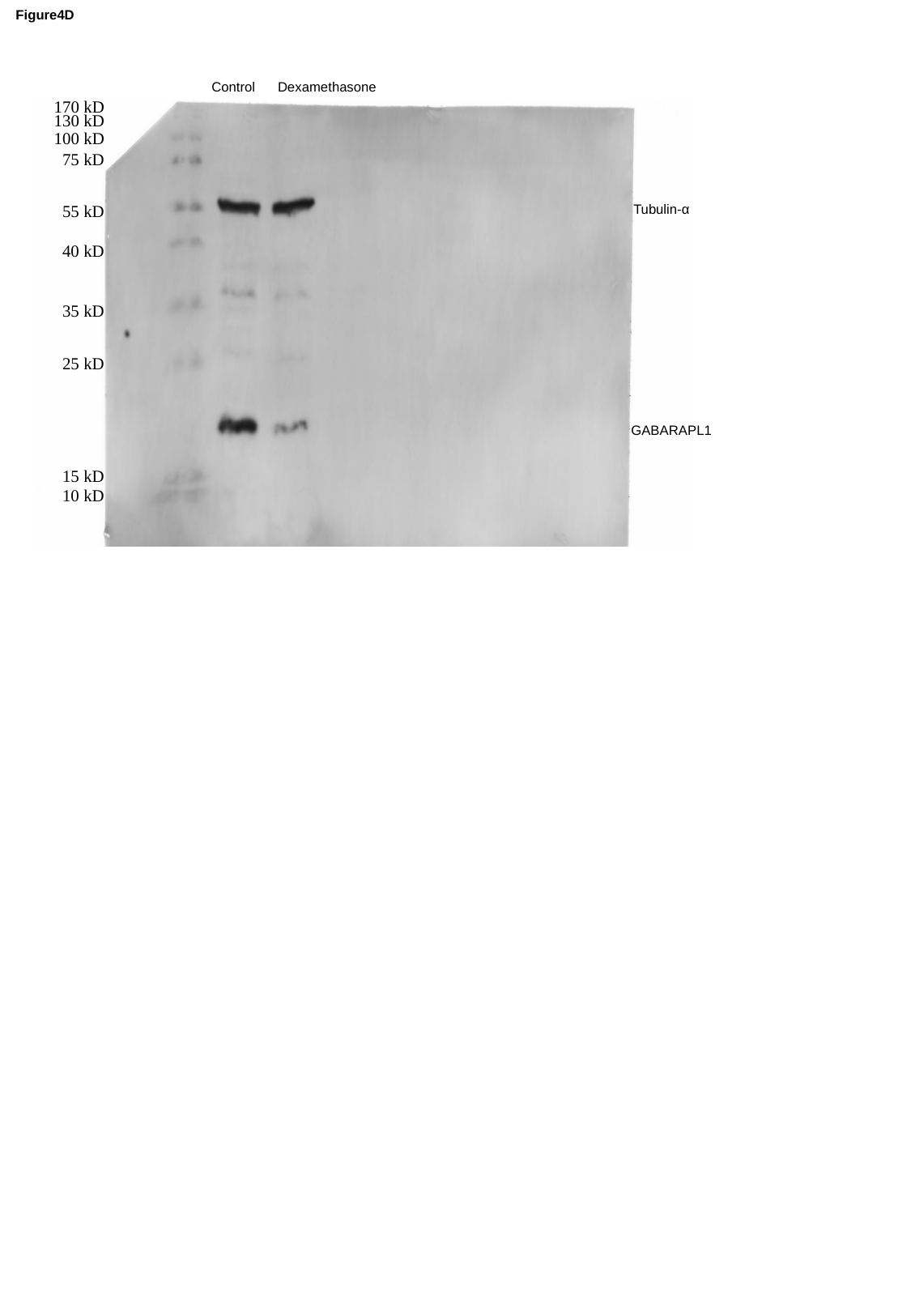

Figure4D
Control
Dexamethasone
170 kD
130 kD
100 kD
75 kD
55 kD
Tubulin-α
40 kD
35 kD
25 kD
GABARAPL1
15 kD
10 kD

## Slide 4
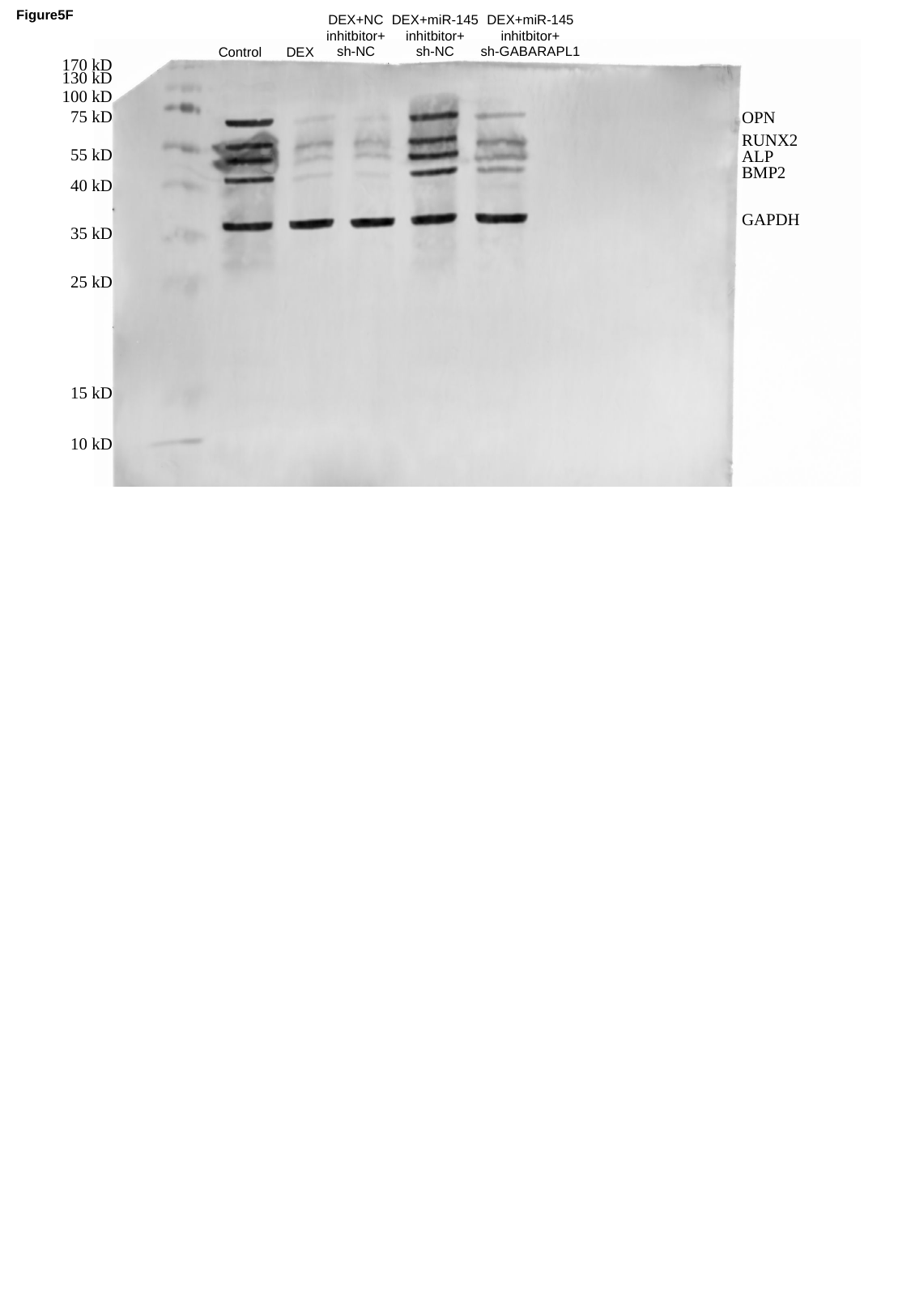

Figure5F
DEX+NC inhitbitor+
sh-NC
DEX+miR-145 inhitbitor+
sh-NC
DEX+miR-145 inhitbitor+
sh-GABARAPL1
Control
DEX
170 kD
130 kD
100 kD
75 kD
OPN
RUNX2
55 kD
ALP
BMP2
40 kD
GAPDH
35 kD
25 kD
15 kD
10 kD
